# Emerging Frontiers in CRISPR-Based Strategies for the Detection and Degradation of Microplastics

**DOI:** 10.3390/life16081261

**Published:** 2026-07-30

**Authors:** Selma Hamimed, Rayane Merazka, Amel Kamah, Fatima Zohra Kamah, Mouna Keroui

**Affiliations:** 1 Molecular and Cellular Biology Laboratory (MCBL), Department of Molecular and Cellular Biology, Faculty of Nature and Life Sciences, University of Jijel, Jijel PC 18000, Algeria; 2 Department of Molecular and Cellular Biology, Faculty of Nature and Life Sciences, University of Jijel, Jijel PC 18000, Algeria; rayanemerazka58@gmail.com (R.M.); amalkamah1@gmail.com (A.K.); kamahfatima@gmail.com (F.Z.K.); mounakeroui@gmail.com (M.K.)

**Keywords:** CRISPR-Cas9, engineered microorganisms, microplastic pollution, biosensors, bioremediation, nucleic acid detection, plastisphere

## Abstract

CRISPR (clustered regularly interspaced short palindromic repeats)-based genome engineering is reshaping how environmental contamination can be interrogated and remediated, offering a level of programmability and specificity that conventional physicochemical workflows seldom match. Microplastics polymer fragments below 5 mm that now pervade virtually every ecosystem are especially difficult to monitor and remove because of their chemical heterogeneity, sub-millimeter size, and capacity to adsorb co-pollutants. This review examines how the molecular logic of CRISPR-Cas systems is being repurposed for two complementary goals: sensitive analytical detection and microbially driven degradation of plastic particles. We first outline the biochemistry of Cas-mediated cis- and trans-cleavage that underpins isothermal, amplification-free biosensing, and then survey direct strategies, in which polymer-binding DNA (deoxyribonucleic acid) aptamers are coupled to Cas12a (CRISPR-associated protein 12a), alongside indirect strategies that read out the molecular stress signatures provoked by microplastic exposure in sentinel organisms and plastisphere communities. On the remediation side, we discuss how targeted editing, CRISPR interference, and rationally assembled microbial consortia enhance enzymatic depolymerization and redirect carbon flux toward valuable bioproducts. By integrating detection and remediation within a single conceptual framework, we identify the principal bottlenecks, aptamer selectivity in complex matrices, reagent stability under field conditions, and host metabolic burden, and outline research priorities for translating these tools from proof of concept toward deployable environmental technologies.

## 1. Introduction

Plastic manufacturing has expanded relentlessly since the turn of the century, and a substantial share of the resulting waste escapes formal collection and treatment [1]. Among the by-products of this mismanagement are microplastics (MPs), synthetic polymer particles smaller than 5 mm whose defining traits are environmental persistence, sub-millimeter dimensions, and a propensity to accumulate in tissues and exert toxicity [2]. These particles enter ecosystems both as primary inputs, including manufactured microbeads and textile fibers, and as secondary fragments generated when larger debris is broken down by mechanical, photochemical, and microbial weathering [3,4].

MPs are commonly categorized by dimension, shape (fibers, fragments, films, and spheres), and polymer chemistry, with polyethylene (PE), polypropylene (PP), polystyrene (PS), polyvinyl chloride (PVC), and polyethylene terephthalate (PET) accounting for most of the material recovered from field samples [5].

Once in the environment, MPs are governed by interacting processes, transport, continued fragmentation, aggregation, and settling, that dictate where they ultimately reside. Their resistance to degradation allows them to persist over long timescales, while their high surface-area-to-volume ratio promotes the sorption of hazardous co-contaminants such as trace metals and persistent organic pollutants. As a consequence, MPs frequently behave as mobile carriers that redistribute toxicants through food webs, compounding their ecological footprint [6].

Humans encounter MPs mainly by ingestion and inhalation, with dermal contact representing a secondary route. Particles have been recovered from drinking water, foodstuffs, and indoor and outdoor air, prompting concern over the consequences of sustained low-level exposure [7]. Following uptake, MPs can cross biological barriers and distribute to multiple organs, where they have been associated with inflammation, oxidative stress, cytotoxicity, and possible interference with endocrine signaling [3,8]. The chronic health burden in humans nevertheless remains poorly resolved and warrants systematic study.

Despite intensifying research, reliable detection, quantification, and removal of MPs continue to pose substantial obstacles. Established analytical routes, optical microscopy and vibrational spectroscopies such as Fourier-transform infrared (FTIR) and Raman, are constrained by cost, throughput, inconsistent protocols, and limited sensitivity toward the smallest particles. In parallel, prevailing remediation options, including physical filtration and chemical oxidation, are often energy-intensive, insufficiently selective, or difficult to reconcile with sustainability goals [9].

These shortcomings have motivated a search for alternatives capable of both finer detection and more sustainable cleanup. Convergent advances in nanomaterials, biosensing, and programmable gene-editing tools are now opening routes to sequence-specific recognition and microbially mediated remediation that were previously out of reach; when paired with regulatory action and circular-economy thinking, such tools could help curb MP release closer to its source [10,11,12]. CRISPR-Cas systems are especially compelling in this regard, because their guide-programmable recognition can be redirected toward almost any molecular target and can report the presence of analytes at very low concentrations [9].

Although CRISPR diagnostics and CRISPR-assisted bioremediation have each advanced rapidly, the two lines of work have largely developed in isolation, and most existing reviews address either detection chemistry or microbial engineering rather than the mechanistic logic that connects them. This separation obscures an opportunity. The same molecular machinery that turns a binding event into an amplified signal can, in another configuration, reprogram the metabolism of plastic-degrading microorganisms. The present review therefore brings detection and remediation into a single framework. We trace the path from the fundamental enzymology of Cas effectors to applied biosensing and to engineered microbial consortia, distinguish direct nucleic-acid-mimicking recognition of polymers from indirect read-outs of biological stress responses, and we examine the specificity limits of each rather than presenting them as interchangeable. Because the field is at an early stage, we frame the synthesis as a forward-looking roadmap: we state explicitly which capabilities are experimentally demonstrated, which are extrapolated from analogous CRISPR systems, and which remain conceptual, and we propose an integrated ‘detect-and-degrade’ architecture that, to our knowledge, has not previously been articulated. A simple technology readiness assessment is applied through a comprehensive pilot feasibility study, laboratory validation, pilot-scale application, and real-world environmental deployment. Detection and processing are treated as complementary, not parallel, processes: the polymer-derived biomarkers and fingerprints recognized by the CRISPR sensor are precisely the signals capable of triggering the CRISPR-designed depolymerization circuit, while the monomers released through the designed depolymerization process provide distinct analytical materials for detection. This interrelationship is illustrated in each thematic section and then formulated into a coherent framework.

## 2. CRISPR Technology: From Gene Editing to Advanced Biotechnology

Originating from a defense system that bacteria and archaea deploy against mobile genetic elements, CRISPR-Cas9 has become a defining tool of modern biotechnology by allowing genetic sequences to be rewritten in a targeted, user-defined manner [13]. In its canonical form, a short guide RNA escorts the Cas9 nuclease to a matching DNA site and triggers a double-strand break, which the host then resolves through its native repair pathways to inactivate, repair, or insert sequence. What began as a method for editing genes has since expanded into therapeutics, agriculture, and molecular diagnostics, encompassing, for example, treatments for inherited disorders such as sickle cell disease [14] and the generation of crops with improved agronomic traits, establishing the platform as a cornerstone technology across the life sciences [15].

### 2.1. The CRISPR-Cas Revolution

The emergence of CRISPR-Cas marked a conceptual turning point in molecular biology, shifting the discipline from reading genetic information to deliberately rewriting it. Adapting a bacterial immune mechanism into a programmable editor [13] placed precise genome modification within reach of essentially any laboratory and across a broad taxonomic range. That advance seeded a wave of more refined tools; base and prime editors, in particular, now enable single-nucleotide substitutions without severing both DNA strands, reducing reliance on error-prone break repair [16]. The downstream applications are far-reaching, extending from curative interventions for previously intractable conditions such as sickle cell disease [14] to the development of climate-resilient crops, and they continue to reshape medicine, agriculture, and basic biological inquiry.

### 2.2. Mechanism of Action of CRISPR-Cas

**Figure 1:**
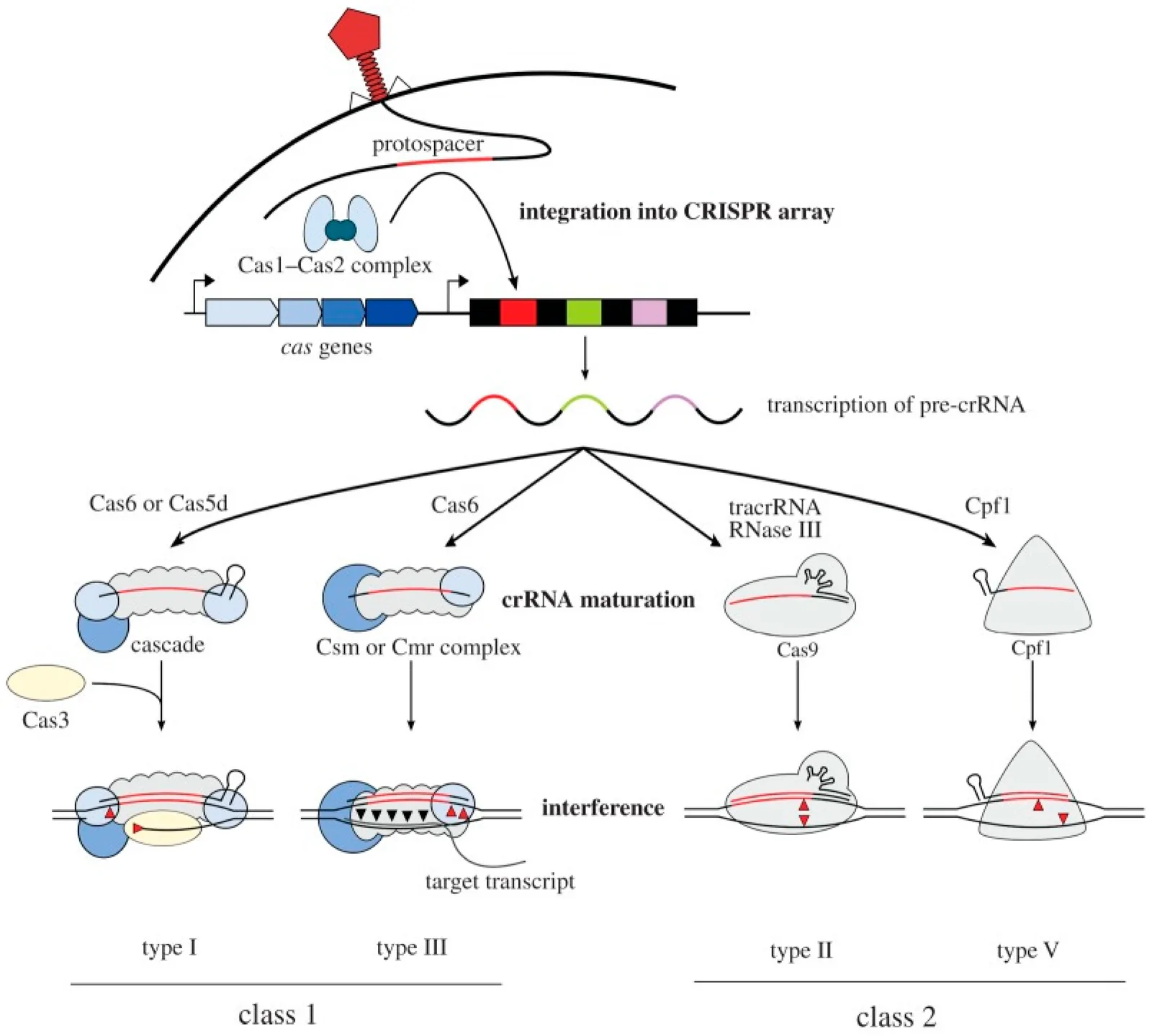
Schematic overview of class 1 and class 2 CRISPR-Cas immune mechanisms. Each system comprises a cas operon (black arrows) and a CRISPR array of identical repeats (black boxes) separated by phage-derived spacers (colored boxes). During adaptation, a segment of invading DNA (the protospacer) is captured by the Cas1–Cas2 complex and integrated into the array. The array is transcribed into a precursor crRNA (pre-crRNA), which is processed by Cas6 in type I and III systems (by Cas5d in type I-C). In type II systems, crRNA maturation requires tracrRNA, RNase III, and Cas9, whereas Cpf1 alone suffices in type V-A. During interference, type I Cascade complexes use crRNA to bind target DNA and recruit Cas3 for degradation; type III-A and III-B systems use Csm and Cmr complexes to cleave DNA (red triangles) and transcripts (black triangles); type II systems rely on a Cas9–tracrRNA:crRNA complex; and type V systems use Cpf1. Adapted from [19].

CRISPR-Cas immunity in prokaryotes proceeds through a multistage process that has since been co-opted for genome editing. In the acquisition phase, fragments of invading viral or plasmid DNA are captured and inserted as “spacers” between the short palindromic repeats of the CRISPR locus, creating a heritable record of prior encounters [17] (Figure 1). Upon a subsequent challenge, the array is transcribed and matured into short CRISPR RNAs (crRNAs) that direct Cas proteins to locate and cut complementary sequences in the invader [18].

In the most widely adopted system, Cas9 relies on a two-RNA arrangement, a crRNA that specifies the target and a *trans*-activating crRNA (tracrRNA) that assists maturation, frequently fused into a single guide RNA (sgRNA). The sgRNA positions Cas9 at a DNA sequence flanked by a protospacer-adjacent motif (PAM), canonically 5′-NGG-3′ for *Streptococcus pyogenes* Cas9 [18]. Target engagement drives conformational rearrangements that activate the enzyme’s two catalytic domains: HNH cleaves the strand paired with the guide, while the RuvC-like domain cleaves the opposite strand, yielding a defined double-strand break (DSB) [20]. Repair of that break supports editing through two main routes—non-homologous end joining (NHEJ), which tends to introduce small insertions or deletions that knock out gene function, and homology-directed repair (HDR), which can install precise edits when a donor template is supplied [21].

### 2.3. Mechanism of CRISPR-Based Detection

**Figure 2:**
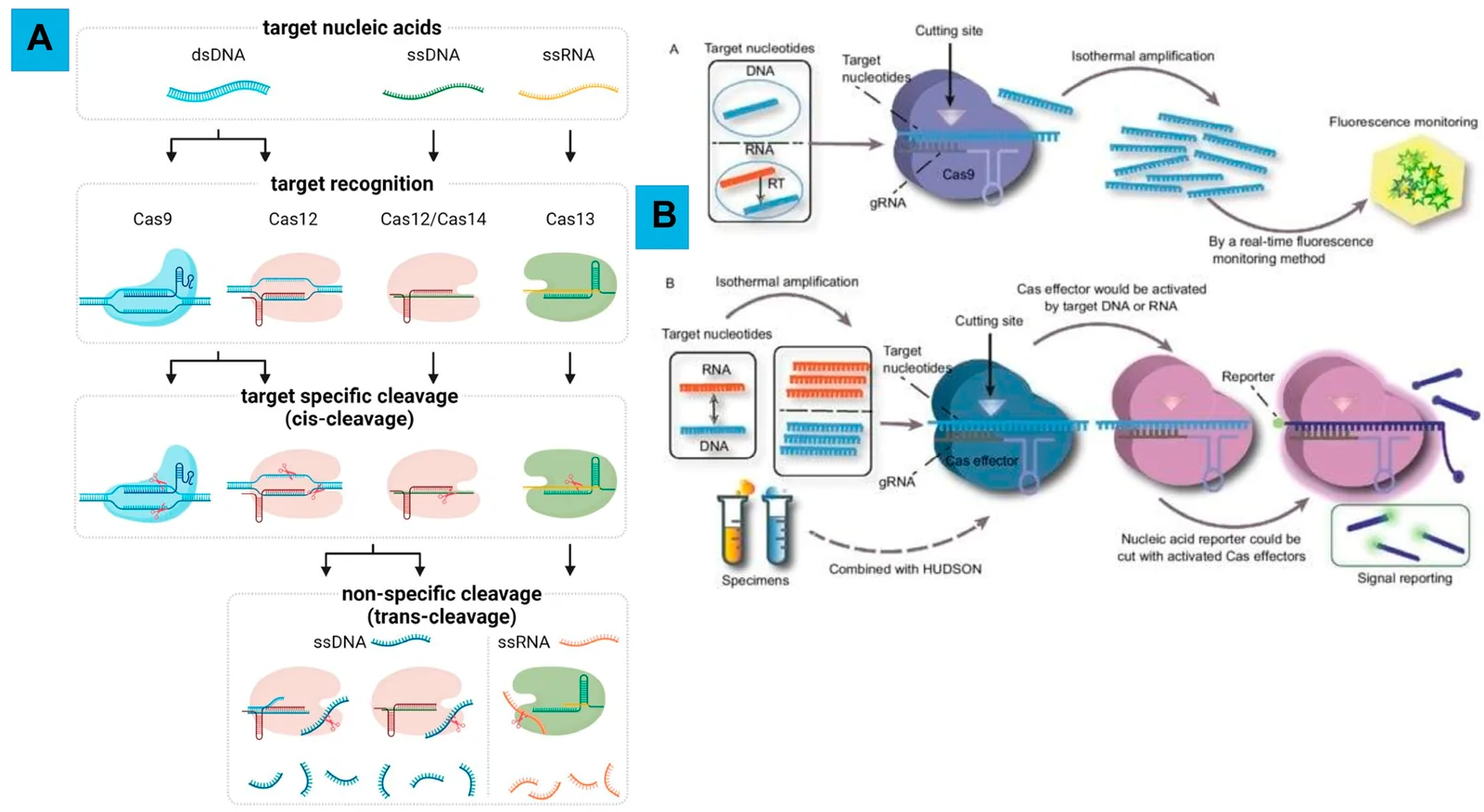
(**A**) Molecular basis of CRISPR-associated nucleic-acid detection. A Cas-gRNA complex first assembles and engages the target nucleic acid, with the effector chosen according to the target type. The ribonucleoprotein then cleaves the bound target (*cis*-cleavage). Whereas Cas9 stops at this step, Cas12, Cas13, and Cas14 additionally cleave nearby non-target nucleic acids (*trans*-cleavage); this collateral activity is the basis of signal amplification in CRISPR diagnostics. Adapted from [24]. (**B**) Mechanism of CRISPR-based detection. (**A**) Cas9-mediated detection relies on guide RNA-directed recognition and cleavage of target nucleic acids, typically combined with amplification and signal generation for sensitive detection. (**B**) Cas12-, Cas13-, and Cas14-based detection exploits target-activated collateral cleavage of reporter molecules, producing measurable signals such as fluorescence or electrochemical responses for rapid and specific target identification. Adapted from [25].

Diagnostic applications exploit a different facet of CRISPR biology. Rather than depending on the site-specific cutting used for editing, detection platforms employ Cas effectors that, once they recognize their target, begin to cleave surrounding nucleic acids indiscriminately—so-called collateral or trans-cleavage activity [22]. The best-characterized effectors of this kind are Cas13a, which acts on RNA, and Cas12a, which acts on DNA; both retain the ability to shred bystander reporter molecules after engaging a matching target sequence [23] (Figure 2).

Operationally, the assay begins with assembly of the Cas effector and its guide RNA. When the complex binds a complementary target, viral RNA, bacterial DNA, or another nucleic-acid biomarker, it switches into a catalytically active state that licenses collateral cleavage [26]. The activated enzyme then severs nearby reporters, typically fluorophore–quencher-labeled oligonucleotides, releasing a measurable fluorescent, colorimetric, or electrochemical signal [27]. This principle underlies platforms such as SHERLOCK (Specific High-sensitivity Enzymatic Reporter unLOCKing), built on Cas13, and DETECTR (DNA Endonuclease-Targeted CRISPR Trans Reporter), built on Cas12, which together can reach attomolar sensitivity and single-nucleotide discrimination without elaborate instrumentation [28].

### 2.4. Advantages of CRISPR Biosensors

CRISPR biosensors combine several properties that are difficult to obtain simultaneously with conventional diagnostics, namely high sensitivity and specificity together with rapid, low-complexity operation. These strengths derive largely from Cas effectors that display collateral cleavage, such as Cas12, Cas13, and Cas14.

In terms of specificity and programmability, the guide RNAs confer single-base resolution, allowing one assay to distinguish closely related sequences, including single-nucleotide polymorphisms (SNPs) and viral variants [22]. Because target choice is dictated solely by the guide sequence, a single platform can be redirected toward DNA, RNA, proteins, or small molecules simply by swapping the guide, yielding a versatile chassis that adapts readily to new analytes.

These systems can register target concentrations in the attomolar (10^−18^ M) range without prior amplification, approaching the performance of PCR [23]. Coupling CRISPR recognition to isothermal amplification chemistries such as recombinase polymerase amplification (RPA) or loop-mediated isothermal amplification (LAMP) shortens time-to-result to roughly 15–60 min, well below culture-based or laboratory PCR workflows.

**Figure 3:**
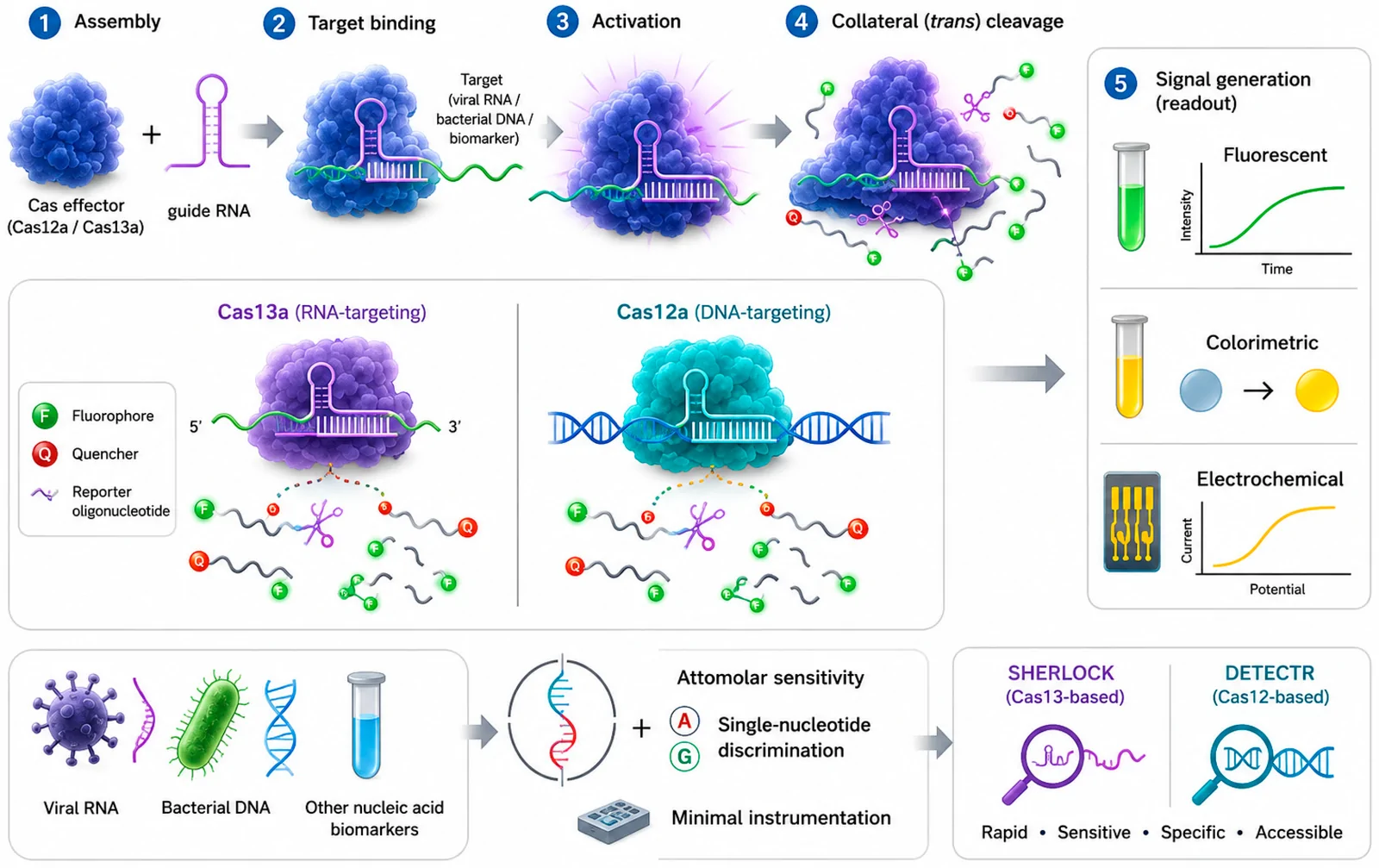
Schematic illustration of mechanism of CRISPR-based nucleic acid detection using Cas13a and Cas12a underlying the SHERLOCK and DETECTR platforms.

CRISPR assays operate at a single temperature and produce outputs fluorescence, color change, or lateral-flow bands, that are easy to interpret, which suits them to settings with limited infrastructure [26]. SHERLOCK and DETECTR have been reduced to paper strips and field kits, broadening access to molecular testing, offering portability and point-of-care compatibility (Figure 3).

The distinct substrate preferences of different Cas enzymes (for instance, Cas12 for DNA and Cas13 for RNA) permit several targets to be interrogated in one reaction [27]. The reagents are stable and inexpensive and require minimal equipment, lowering per-assay costs relative to established methods while preserving accuracy.

### 2.5. Mechanism of CRISPR-Based Editing and Therapeutics

**Figure 4:**
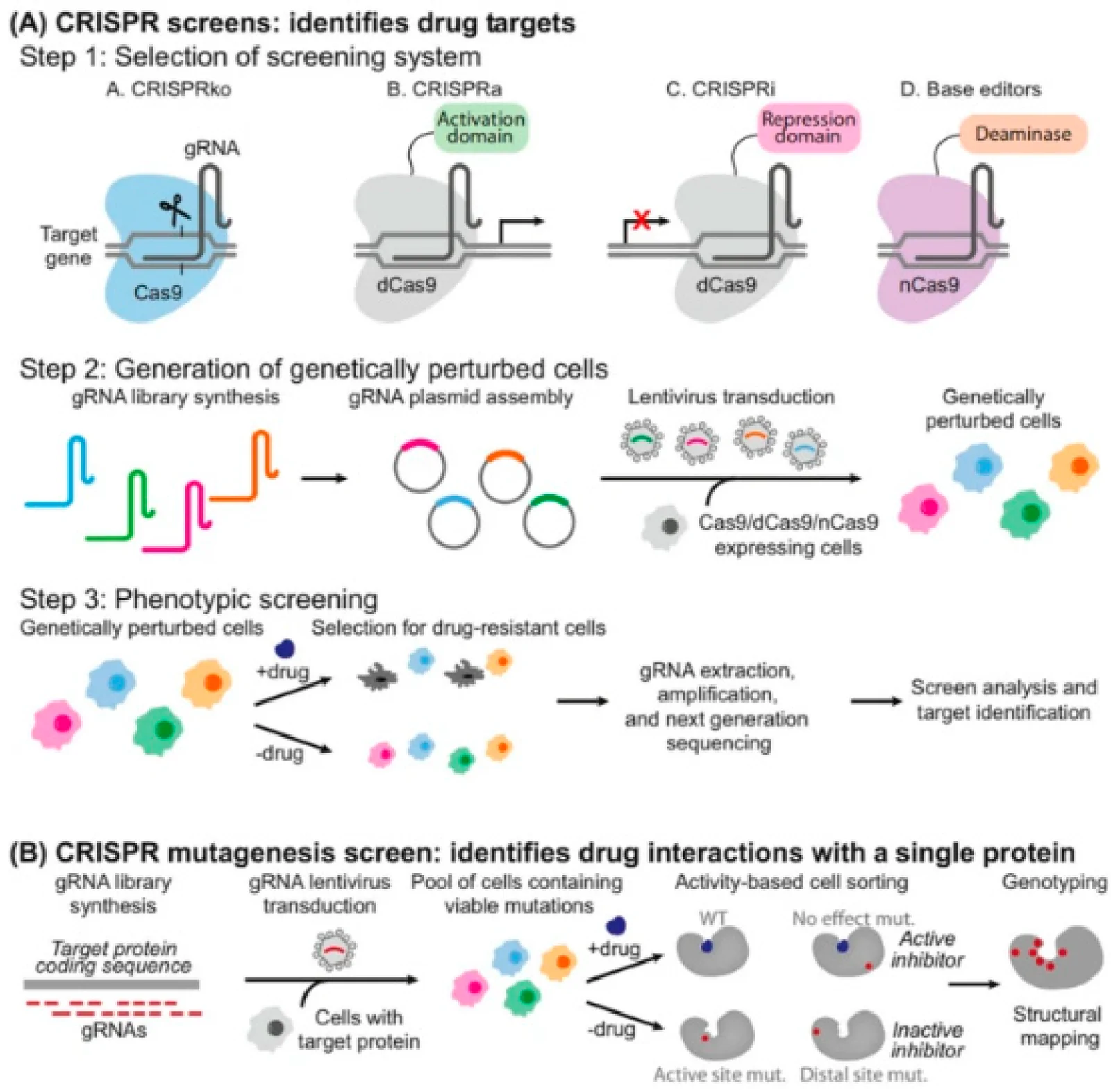
CRISPR screening and mutagenesis workflows. (**A**) Screening begins with selection of a system CRISPRko (Cas9-mediated disruption via frameshift or premature stop codons), CRISPRa (dCas9 fused to activation domains such as VPR or VP64), CRISPRi (dCas9 fused to repressors such as KRAB), or base editing (cytosine or adenine deaminases for break-independent point mutations). A gRNA library is introduced into cells, followed by selection, gRNA amplification, and next-generation sequencing to identify target genes. (**B**) Mutagenesis screening uses a gRNA library that generates in-frame mutations in a target coding sequence; after transduction and drug treatment, activity-based sorting enriches mutation-bearing cells, and escape mutants are identified by deep sequencing. Adapted from [29].

CRISPR-based therapeutics correct disease-causing mutations directly at the DNA level and, more broadly, illustrate the editing modalities that are later applied to microbial engineering for remediation. Clinically, the approach builds on the core Cas9 system but adds safeguards needed for use in patients. Delivery of the components is the first step, achieved with viral vectors such as adeno-associated virus or lentivirus, or with non-viral carriers such as lipid nanoparticles. Inside the cell, the guide RNA steers Cas9 to the pathogenic locus, where a precisely placed double-strand break recruits the cell’s repair machinery to effect the intended change (Figure 4) [14].

The two repair routes are leveraged for distinct outcomes. Where the goal is to switch a gene off, the indel-generating NHEJ pathway is used deliberately, as in sickle cell disease and β-thalassemia, where disrupting *BCL11A* reactivates fetal hemoglobin. Where precise correction is required, HDR is engaged together with a donor template carrying the correct sequence [14]. More recent base- and prime-editing systems convert one base into another without inducing double-strand breaks, markedly limiting off-target liabilities and proving especially useful for the point mutations underlying certain metabolic and muscular disorders [16]. These modalities are deployed either ex vivo-editing a patient’s cells, such as hematopoietic stem cells or T cells, in culture before reinfusion, or in vivo, by administering the editing machinery directly to reach tissues that are otherwise difficult to access.

## 3. CRISPR for Microplastic Bioremediation

Beyond clinical and agricultural uses, the same editing toolkit can be turned toward environmental cleanup by upgrading the catalytic capabilities of microorganisms that act on plastics. Precise genome editing, Cas9-based approaches in particular, provides a controlled means of reinforcing the metabolic functions that drive microplastic breakdown, opening realistic avenues for biologically based remediation.

It is important to place CRISPR technology within a broader toolkit in microbial and enzyme engineering, as many of the most significant advances in plastic biodegradation have emerged from complementary approaches, such as metagenomic and functional screening of novel hydrolases [30], adaptive in vitro evolution, structure-guided and machine-learning-assisted evolution, and systems-level metabolic modeling.

**Figure 5:**
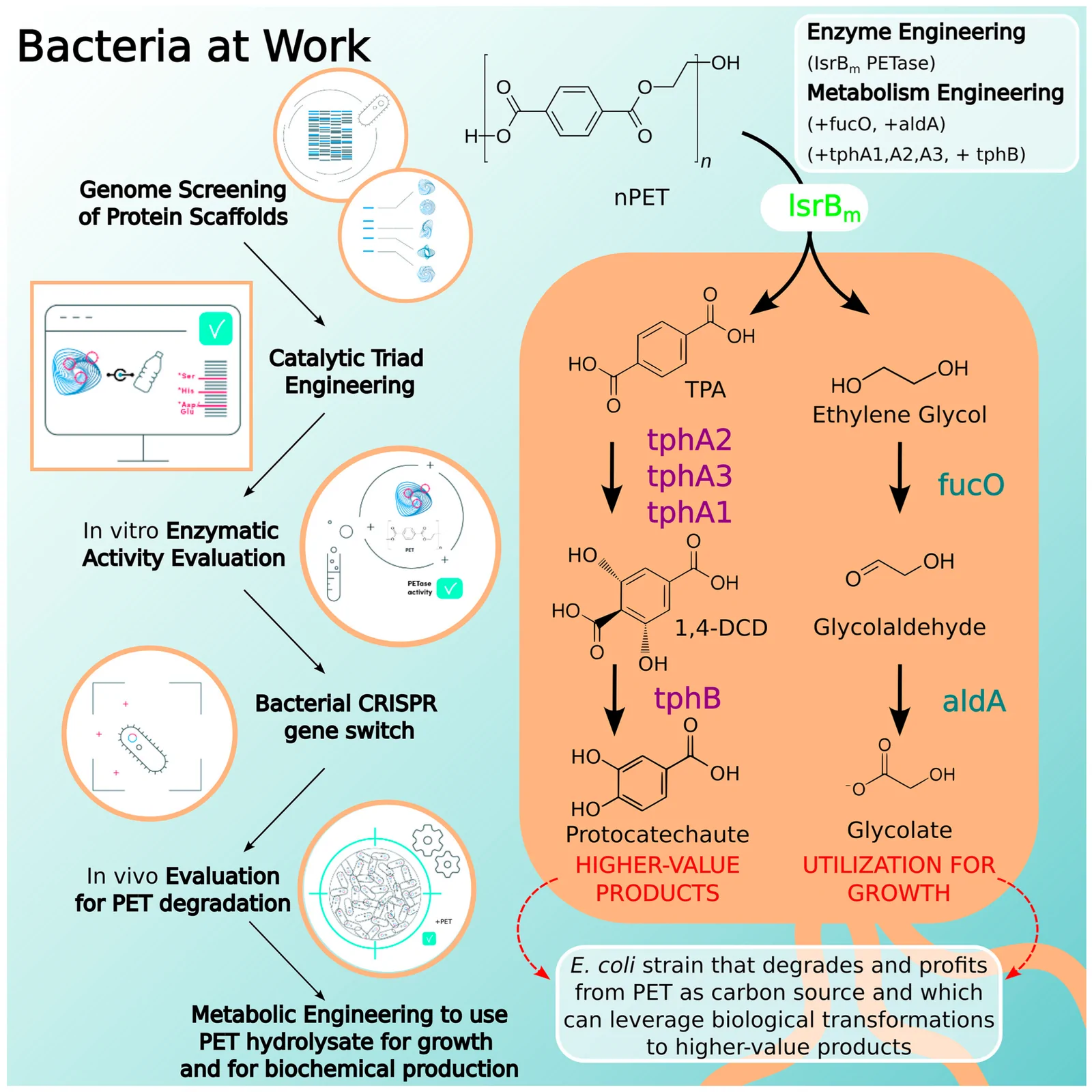
Integrated workflow for reprogramming *Escherichia coli* to degrade and valorize PET without foreign DNA. Adapted from [33].

FAST-PETase, for instance, was engineered through a structure-based machine-learning campaign [31]: differing from wild-type PETase by only five mutations, the variant exhibits superior catalytic activity across the 30–50 °C range relative to both the wild-type enzyme and all previously reported mutants. Comparable gains have been achieved by orthogonal strategies, as proved by HotPETase, which was derived through automated high-throughput directed evolution platform [32]. A recent proof-of-concept study exemplifies how these modules converge within a single microbial chassis (Figure 5). Rather than introducing heterologous depolymerases, native *Escherichia coli* BL21(DE3) proteins were selected in silico from the genome and computationally repurposed to acquire artificial PETase activity while preserving their endogenous function, after which the corresponding loci were edited in place by CRISPR/Cas9 rather than by insertion of foreign DNA. It showed that the strategy is transferable across diverse genomes and microbial framework, and it illustrates how in silico enzyme discovery, CRISPR-mediated implementation, and metabolic engineering can be integrated to advance both PET biodegradation and upcycling [33].

### 3.1. Engineering Microorganisms for Plastic Degradation

At the heart of the “PlastiCRISPR” concept is the use of guide RNAs designed to recognize defined genomic loci in a target microorganism. Once the guide directs Cas9 to the chosen site, the nuclease introduces a double-strand break that permits sequence to be inserted, removed, or altered with precision; in this way, strains can be engineered to elevate production of the enzymes that catalyze polymer breakdown [34]. Cellular repair via NHEJ or HDR then fixes the intended modification in the genome [35,36]. Representative engineered microorganisms, together with their substrates, pathways, and products, are compiled in Table 1.

Several studies have already used CRISPR to retool bacteria and fungi for stronger plastic degradation. For example, *Escherichia coli* and *Pseudomonas putida* have been edited to express PET-hydrolyzing enzymes, raising PET-degradation efficiency [43]. In *E. coli*, engineering has produced fusion constructs that join PETase with MHETase or with laccases to improve substrate binding, thermostability, and activity under industrially relevant conditions; in one design, a whole-cell biocatalyst displayed a PETase variant (FAST-PETase) alongside carbohydrate-binding module 3 (CBM3) on the cell surface while MHETase was expressed in the cytoplasm, constituting a bacterial enzyme-cascade system for PET breakdown [44]. Such strains sustain PET degradation even under stressors such as elevated temperature or fluctuating pH [43].

When directly comparing the engineered structures in Table 1, distinct trade-offs emerge rather than a single superior platform. *Ideonella sakaiensis* remains the paradigmatic native PET assimilator, encoding a complete PETase–MHETase route, yet its slow growth, mesophilic optimum, and comparatively immature genetic toolbox limit scalability. *Pseudomonas putida* offers exceptional solvent and oxidative-stress tolerance together with a versatile central metabolism, making it an attractive monomer-valorizing framework, but it lacks native depolymerases and therefore depends on heterologous enzyme expression. *Escherichia coli* is the most genetically tractable host and supports the highest reported surface-display and fusion-enzyme, although its limited environmental robustness confines it largely to bioreactors. *Actinomycetes* such as *Streptomyces* and *Rhodococcus* spp. provide strong secretory capacity and resistance to desiccation and oligotrophic stress that suit in situ application, but slower growth and less-developed editing tools currently restrict throughput. Importantly, reported degradation efficiencies are difficult to settle across studies because substrate crystallinity (amorphous versus semicrystalline PET), particle size, temperature, and assay duration are rarely standardized.

### 3.2. CRISPR-Mediated Metabolic Engineering

CRISPR offers fine control over the metabolic and regulatory circuitry of microbial hosts. Tuning promoters, adjusting ribosome-binding sites, and assembling synthetic gene circuits make it possible to govern when and how strongly plastic-degrading enzymes are produced, constitutively, on induction, or in response to defined environmental cues [45]. CRISPR interference (CRISPRi) has been applied in *P. putida* to throttle competing routes such as β-oxidation, channeling carbon instead toward polyhydroxyalkanoate (PHA) synthesis from plastic-derived monomers; these interventions embody the iterative design-build-test-learn cycle of synthetic biology aimed at maximizing conversion of plastic into value-added products [46]. A caveat is that forcing expression of enzymes such as PETase and cytochrome P450 imposes a metabolic load by diverting cellular resources, which can slow growth and depress product yields [47,48].

Importantly, CRISPR engineering serves not only to reduce plastic pollution but also to manufacture bio-based plastics from renewable feedstocks, supporting a low-carbon circular bioeconomy. Using Cas9-mediated editing, *E. coli* was reprogrammed by overexpressing the *pntAB* operon and deleting four by-product-forming genes (*pflB*, *ldhA*, *adhE*, and *fnr*); the resulting strain, HR002, showed faster growth, higher PHA content, fewer by-products, and elevated intracellular acetyl-CoA [49]. CRISPR-guided enzyme engineering has likewise improved the thermostability and turnover of PETase, speeding depolymerization of PET into recyclable monomers [50], while a near-quantitative editing strategy in *Halomonas bluephagenesis* substantially increased incorporation of key monomers into PHA copolymers [51]. Yet, these engineered whole-cell biocatalysts, including the FAST-PETase surface-display system, remain laboratory-scale demonstrations and have not been evaluated at pilot scale or under authentic environmental conditions.

### 3.3. Constructing Synthetic Microbial Consortia

A growing theme within synthetic biology is the deliberate construction of microbial consortia [52], defined communities of two or more strains or species (often two to three) comprising bacteria, fungi, or both [53,54]. Interest in these designed assemblies stems from their utility in biosynthesis and bioremediation [53]: by combining complementary metabolic capabilities, consortium members can accomplish polymer breakdown that no single organism could achieve alone [54].

CRISPR-Cas tools add a further dimension by enabling genetic or metabolic tailoring of individual members. An effective synthetic consortium ideally exhibits stable interdependence among partners, reduced competition for substrates, and increased flux toward the desired output [53]. In such designs, engineered partners can divide labor, one strain hydrolyzing the polymer while another converts the released monomers into biofuels, bioplastics, or other materials [55]. The same logic extends to contaminated agricultural soils, where purpose-built consortia degrade MPs and residual mulch films and, over time, improve soil quality and crop performance [43]. CRISPR-Cas can additionally be used to prune or rebalance communities: in *E. coli*, the type I-E system has been shown to discriminate and remove closely related strains, with community composition tunable by varying the delivered CRISPR RNAs [56]. It should be emphasized that consortia offer ecological robustness, metabolic complementarity, and resilience under the fluctuating temperature, pH, and nutrient conditions of real environments, and they distribute metabolic burden across members so that no single strain must carry the entire depolymerize, assimilate, and valorize pathway; where suitable partners already exist in nature, they also demand comparatively little genome engineering. In practice when combined, with CRISPR serving as the tool that installs or optimizes a function within an otherwise self-organizing community. Table 2 contrasts the relative strengths and applicability of Cas9-based editing with the synthetic-consortium approach for plastic degradation.

### 3.4. Enhancing Biofilm Formation on Plastic Surfaces

Biofilm development on plastic debris unfolds in stages, beginning with attachment of pioneer colonizers and continuing with recruitment of secondary taxa [59]. As the community matures, it organizes into a structured architecture in which distinct populations occupy defined microhabitats [60], embedded within a matrix of extracellular polymeric substances (EPS) that incorporates extracellular DNA and fluid channels [61]. The EPS network holds the biofilm together and confers mechanical resilience, with viscoelastic behavior arising from interactions between the matrix and bacterial aggregates [62]; this organization aids nutrient distribution and enhances both physiological and genetic robustness relative to free-living cells [63].

**Figure 6:**
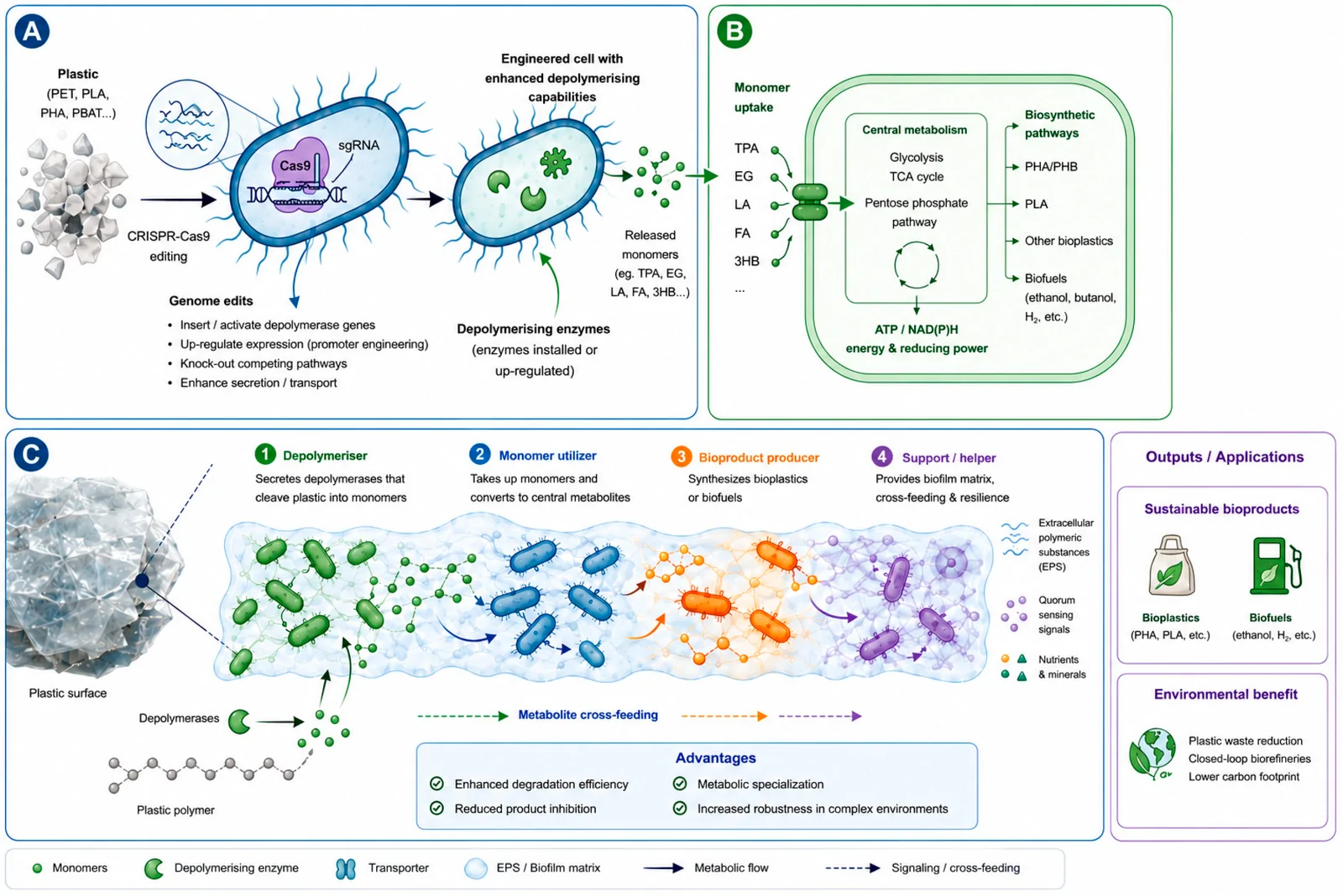
Proposed overview of the PlastiCRISPR strategy for engineering microbial plastic degradation and bioproduct synthesis. (**A**) CRISPR–Cas9 genome engineering enhances the plastic-degrading capacity of a microbial framework by introducing or activating depolymerase genes, up-regulating their expression, eliminating competing pathways, and improving enzyme secretion or substrate transport. The engineered microorganisms efficiently depolymerize diverse plastics (e.g., PET, PLA, PHA, and PBAT), releasing assimilable monomers such as terephthalic acid (TPA), ethylene glycol (EG), lactic acid (LA), fatty acids (FA), and 3-hydroxybutyrate (3HB). (**B**) The released monomers are imported into the cell and redirected through central metabolic pathways (glycolysis, the tricarboxylic acid (TCA) cycle, and the pentose phosphate pathway) to generate ATP and reducing equivalents (NAD(P)H), which fuel the biosynthesis of value-added products, including polyhydroxyalkanoates (PHA/PHB), polylactic acid (PLA), other bioplastics, and biofuels (e.g., ethanol, butanol, and H_2_). (**C**) Within the plastisphere biofilm, a synthetic microbial consortium distributes complementary functions among specialized members: (1) depolymerizers secrete extracellular enzymes to cleave plastic polymers, (2) monomer utilizers assimilate degradation products into central metabolism, (3) producer strains convert intermediates into bioplastics or biofuels, and (4) Co- microorganisms enhance biofilm formation, metabolic cross-feeding, quorum sensing, and community resilience.

Several CRISPR-Cas systems, notably types I and II, have been found to influence biofilm formation directly by modulating quorum-sensing circuits that coordinate adhesion and structural assembly [63,64,65]. Precise genome editing, again principally Cas9, has enabled microbial genomes to be furnished with genes encoding MP-degrading enzymes. Along these lines, a strain of *Pseudomonas aeruginosa* was engineered to overproduce adhesive EPS, strengthening biofilm formation on plastic surfaces and improving its capacity to capture MPs [9]. Such examples show how combining genetic engineering with controlled biofilm formation can advance MP remediation. Figure 6 illustrates a proposed PlastiCRISPR strategy for engineering microbial plastic degradation and bioproduct synthesis.

## 4. CRISPR for Microplastic Detection

Detection and remediation are not independent endeavors. The same plastisphere biomarkers and polymer-derived molecular signatures that a CRISPR sensor is programmed to recognize can also serve as the inducing signals that gate a CRISPR-engineered degradation circuit; conversely, the monomers released by engineered depolymerization (e.g., terephthalic acid and ethylene glycol) constitute characteristic analytes for detection.

### 4.1. Indirect Detection Strategies

Indirect CRISPR-based detection of MPs does not target the polymer itself; instead, it reads molecular consequences of MP exposure, biological responses or characteristic nucleic-acid sequences. These schemes marry biomarker discovery with Cas effectors such as Cas12 and Cas13, exploiting their specificity and collateral-cleavage-driven signal amplification for nucleic-acid targets [66]. In practice, a Cas12 or Cas13 effector is programmed with a guide complementary to a chosen sequence; binding activates *trans*-cleavage of labeled reporters, fluorescent probes or lateral-flow substrates, producing a quantifiable signal [67].

#### 4.1.1. Targeting Biological Markers of Microplastic Exposure

MPs are well documented as pro-oxidants [68,69,70,71]. Much of their toxicity in exposed organisms traces to oxidative stress driven by surplus reactive oxygen species (ROS), whose accumulation sets off downstream responses including stress signaling, apoptosis, and inflammation [72].

This mechanism is supported across taxa. In *Drosophila melanogaster*, polyethylene MPs sharply increased stress-response transcripts such as Hsp70Bc, p53, and apoptosis-associated genes, consistent with oxidative stress and DNA-damage responses [73]. In bacteria, high PET loading (3%) raised catalase activity across four species, indicating reinforced antioxidant defenses [3]. In the freshwater crustacean *Daphnia magna*, exposure to PE and PS MPs produced epigenetic changes and shifted gene expression and stress-related genes (e.g., *ecr-b*) and downregulating reproductive genes (e.g., v1), revealing the complexity of the molecular response [74]. Aquatic and soil microorganisms exposed to PS and PE likewise show elevated ROS and characteristic transcriptomic signatures, nominating candidate RNA biomarkers for detection assays [75].

Such stress-responsive transcripts can serve as surrogate indicators of MP exposure. A Cas13-based sensor programmed against a specific messenger RNA would, upon binding, cleave neighboring RNA reporters to yield a fluorescent or electrochemical read-out, conferring high specificity and sensitivity for stress-associated RNA biomarkers [76].

#### 4.1.2. Detecting Stress-Response Genes in Bioindicator Organisms

In aquatic settings, MPs create new surfaces for colonization, giving rise to biofilm communities collectively termed the plastisphere. These assemblages are compositionally distinct and are frequently enriched in mobile genetic elements such as antibiotic-resistance genes, reflecting the altered microbial ecology that MP surfaces impose [77].

Algae and bacteria exposed to MP leachates show disturbances in photosynthesis-related genes, membrane-integrity transcripts, and detoxification pathways, stress signatures that can be quantified by CRISPR-based nucleic-acid detection. Although such changes have traditionally been profiled by transcriptomics, pairing this knowledge with Cas12/Cas13 platforms could deliver rapid, sequence-specific assays [10]. Recent developments further allow several transcripts to be monitored at once in complex samples: microfluidic devices integrated with CRISPR assays can interrogate panels of stress-related RNAs in a single reaction, enabling higher-throughput surveillance of exposure biomarkers [78]. Cas13a-based sensors, for instance, can be tuned to detect such transcripts, or signature 16S rRNA sequences from plastisphere bacteria, so that differential expression or the presence of plastisphere-associated nucleic acids becomes an indirect proxy for MP contamination [79].

#### 4.1.3. Identifying DNA/RNA Signatures from Plastic-Associated Communities

Genome-editing tools have been applied widely across plants, animals, and microorganisms to drive expression of selected genes [9]. The progression from zinc-finger proteins and transcription activator-like effector nucleases to CRISPR/Cas9 has made genetic manipulation increasingly tractable [80,81], supporting targeted loss- and gain-of-function studies that adjust the expression of multiple genes at once.

This capability can be directed at installing genes for MP-degrading enzymes, including PET hydrolase, dehalogenase, esterase, depolymerase, and laccase. Notably, three distinct CRISPR sequences were identified in *Streptomyces albogriseolus* LBX-2, where oxygenase is central to polyethylene degradation, marking it as a promising engineering chassis [82].

Although Cas effectors recognize nucleic acids rather than the polymers themselves, they can be aimed at DNA/RNA signatures tied to plastic particles. Plastisphere DNA, including particular 16S rRNA sequences and mobile genetic elements such as antibiotic-resistance genes, has been cataloged across diverse MP types and habitats, revealing plastisphere-specific communities and gene enrichment on MP surfaces [83]. Work on *Streptococcus anginosus* SK52, in which expression of Cas9 alters tolerance to environmental stresses such as UV, hydrogen peroxide, and heat, illustrates how the presence or absence of stress-response genes can be probed in a bioindicator [84]. Cas13a RNA sensors combined with reverse transcription and isothermal amplification (e.g., RT-RPA) provide a route to detecting such stress genes [66].

### 4.2. Direct Detection Strategies

It is important to delineate what has actually been demonstrated, because direct CRISPR-based detection of microplastics remains in its infancy. To date, only a small number of primary studies report quantitative performance for CRISPR-based microplastic assays (Table 3). The most complete demonstration couples a polymer-specific DNA aptamer to a split-guide CRISPR/Cas12a system: recognition of polyvinyl chloride or polystyrene particles triggers a cascade strand-displacement reaction and collateral cleavage of a G-quadruplex reporter immobilized on a gold electrode, yielding limits of detection in the tens-of-nanograms-per-milliliter range [85]. A subsequent study integrated a CRISPR colorimetric aptasensor with smartphone imaging and deep-learning classification to enable visual prediction and selective recycling of PV and PS MPs [86]. A colorimetric hemin-aptamer DNAzyme combined with the trans-cleavage activity of Cas12a exhibited high selectivity ranging from 10^–2^ to 10^3^ μg mL^−1^ and low detection limits of 3.1 ng mL^−1^ for PVC and 3.7 ng mL^−1^ for PS.

When evaluated in comparison with each other rather than separately, the reported CRISPR/Cas12a microplastic sensors show a clear trade-off between sensitivity and ease of deployment. The split-gRNA electrochemical aptasensor provides a robust, label-free read-out (LOD 45 ng mL^−1^ for PS and 37 ng mL^−1^ for PVC), but its electrode-immobilized reporters are susceptible to fouling by humic substances and proteins in environmental matrices. Electrochemiluminescent designs using Alq_3_@ZIF-8 emitters improve sensitivity to roughly 15–20 ng mL^−1^ and, when coupled to rolling-circle amplification and a laser-engineered cathodic emitter, reach 0.20 ng mL^−1^ over a wide linear range; this gain, however, is accompanied by multi-step workflows and specialized instrumentation that reduce field portability. Conversely, the colorimetric aptasensor integrated with smartphone imaging and deep-learning classification is the most readily deployable and even enables visual sorting for selective recycling, but at the cost of higher detection limits and greater sensitivity to ambient lighting and sample color. Critically, selectivity against chemically similar polymers and performance in genuinely complex matrices (natural waters, sediment eluates, biological digests) have been benchmarked in only a minority of these reports; the platform best suited to a given application therefore depends as much on the sample matrix and required throughput as on nominal LOD.

#### 4.2.1. Engineering Specificity for Plastic Polymers

**Figure 7:**
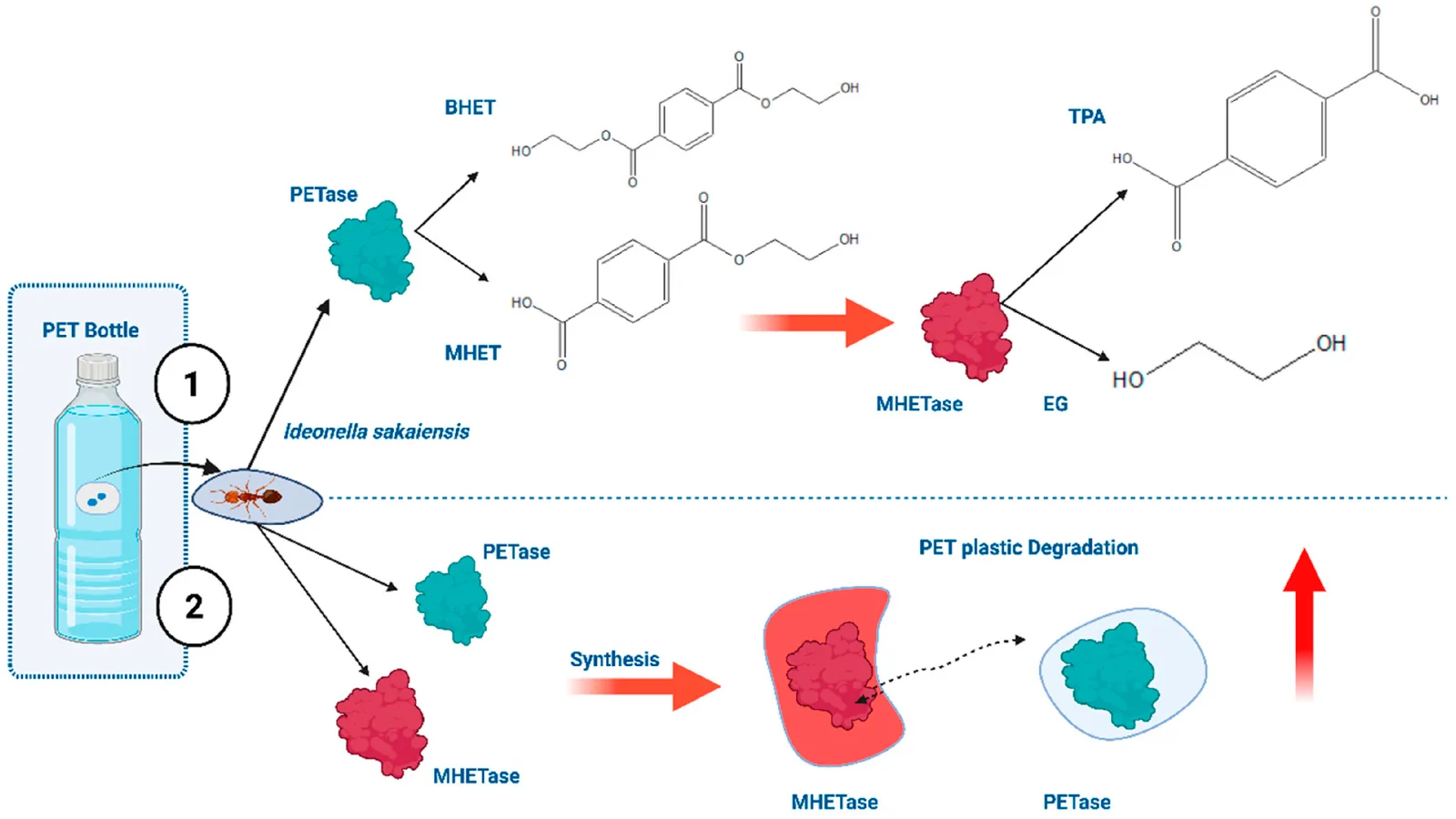
Enzyme-mediated polymer recognition of PET microplastics. PETase and MHETase catalyze the sequential hydrolysis of PET into BHET, MHET, terephthalic acid (TPA), and ethylene glycol (EG). The formation of these characteristic degradation products enables polymer-specific recognition, providing a biochemical basis for the development of advanced biosensing and CRISPR-assisted microplastic detection technologies. Adapted from [43].

While CRISPR technologies have been widely explored in the context of microbial plastic degradation improvement via genome engineering, their importance in microplastic analysis is growing in the development of highly selective recognition systems for polymer-specific detection. Using CRISPR, the microorganisms *Ideonella sakaiensis*, *Escherichia coli* and *Pseudomonas putida* have been engineered to over-express PETase, MHETase and leaf-branch compost cutinase (LCC), enzymes that have strong substrate preferences towards PET (Figure 7). Engineered biorecognition elements produce polymer-specific degradation intermediates, such as terephthalic acid and ethylene glycol, which can act as unique molecular fingerprints for direct identification of microplastics from PET. CRISPR-based optimization of enzyme expression, secretion, and substrate-binding domains raises catalytic affinity for polymer surfaces. This, in turn, improves discrimination among chemically similar plastics. These advances lay the groundwork for next-generation biosensing platforms where polymer-selective enzymatic interactions are coupled to electrochemical, optical, or CRISPR-based signal transduction systems. Crucially, unlike the traditional spectroscopic methods that rely on physicochemical signatures, CRISPR-enabled biorecognition offers a molecular-level approach that can distinguish the target polymers by their unique biochemical transformation pathways. This emerging paradigm offers improved selectivity for polymer identification and may enable the development of rapid, field-deployable tools for the direct detection and characterization of environmentally relevant microplastics [43].

#### 4.2.2. DNA Aptamer-Based Polymer Recognition

DNA aptamers have been developed as highly selective molecular recognition elements for the detection of microplastics, especially PVC and PS, because of their capacity to recognize specific surface physicochemical features of polymer particles [90]. In CRISPR-assisted platforms, target binding by a polymer-specific aptamer induces conformational changes or strand-displacement reactions that generate an activator for Cas12a. Upon activation, Cas12a exhibits strong collateral trans-cleavage activity against single-stranded DNA reporters immobilized on the surface of an electrode, resulting in an amplified electrochemical signal transduction. Coupling aptamer-based target recognition with CRISPR/Cas12a enzymatic signal amplification dramatically improves analytical sensitivity and specificity, permitting the detection of trace levels of MPs in complex environmental samples [91].

#### 4.2.3. Toward Allosteric Cas Proteins for Plastic and Microplastic Leachates

Future biosensors for plastics and their leachates are likely to draw on computational protein design to create programmable, ligand-responsive Cas variants. Allosteric CRISPR activators are more suitable than conventional CRISPR diagnostics in the context of plastic leachates, because they can convert non-nucleic-acid binding events into Cas12a activation, while standard CRISPR diagnostics are optimized for direct nucleic-acid targets. The tradeoff is that allosteric systems greatly expand target scope but generally do not yet match the ultrasensitive, highly mature performance of the best nucleic-acid CRISPR assays [92].

## 5. Mechanistic of an Integrated CRISPR-Based Detect-and-Degrade Architecture for Microplastics

Herein, we articulate the explicit molecular logic by which the output of a CRISPR sensing module can be made to govern the input of a CRISPR-engineered degradation module, thereby converting two independent demonstrations into a closed, stimulus-responsive system. A genuine detect-and-degrade system must satisfy three sequential mechanistic requirements: (i) a molecular recognition event specific to the target polymer or to a polymer-correlated biomarker must be converted into a discrete biochemical signal; (ii) that signal must be propagated and amplified with sufficient gain to cross an activation threshold; and (iii) the supra-threshold signal must be coupled to the transcriptional or post-translational activation of a depolymerizing enzymatic cascade, ideally with negative feedback that attenuates degradative gene expression once the substrate is consumed. The conceptual novelty is therefore not the invention of a new enzyme but the recognition that the collateral-cleavage, conducted by Cas12 and Cas13, and transcriptional-control modalities of the CRISPR toolbox are mutually composable into a feedback circuit, an architecture that to our knowledge has not previously been articulated for environmental microplastic management.

The starting point of the architecture is the conversion of a non-nucleic-acid binding approach onto the physiochemically distinctive surface of MPS. Mechanistically, this is achieved by partitioning the molecular recognition function from the catalytic function: the aptamer is engineered as a structure-switching element whose ligand-bound and ligand-free states differ in their capacity to liberate, expose, or reconstitute a CRISPR-activating nucleic acid. In the split-activator design, polymer binding triggers a toehold-mediated strand-displacement cascade that releases a single-stranded DNA activator complementary to the Cas12a crRNA spacer; hybridization of this activator to the crRNA–target heteroduplex completes the PAM-proximal seed pairing required to relieve the autoinhibitory conformation of the RuvC domain. The resulting allosteric reorganization unleashes indiscriminate trans-cleavage of single-stranded DNA reporters. This non-stoichiometric relationship between one binding event and many cleavage events constitutes the primary signal-amplification stage and is the biochemical reason CRISPR transduction can reach the tens-of-nanograms-per-milliliter detection limits documented for current microplastic aptasensors.

**Figure 8:**
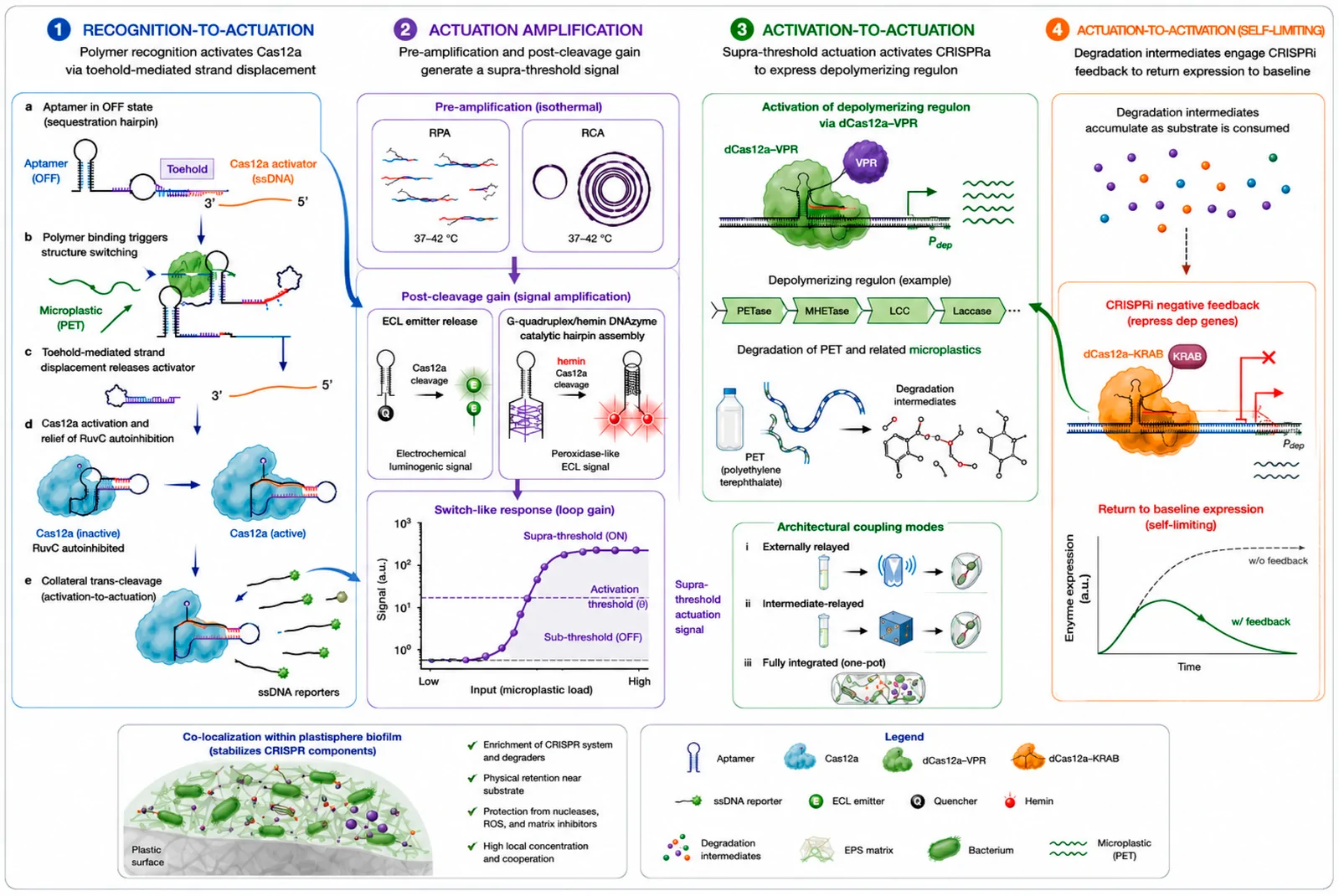
Mechanistic schematic of the proposed integrated CRISPR-based detect-and-degrade architecture for microplastics. Polymer recognition by a structure-switching DNA aptamer triggers toehold-mediated strand displacement that releases a Cas12a-activating single-stranded DNA, relieving RuvC autoinhibition and unleashing collateral trans-cleavage (recognition-to-signal). Isothermal pre-amplification (RPA/RCA) and post-cleavage gain stages (ECL emitter release; G-quadruplex/hemin DNAzyme) raise loop gain to a switch-like threshold (signal propagation). The supra-threshold signal gates dCas12a-VPR-mediated transcriptional activation of a depolymerizing regulon (PETase, MHETase, LCC, laccase) via externally relayed, intermediate-relayed, or fully integrated coupling (signal-to-actuation). Accumulating degradation intermediates drive a CRISPRi negative-feedback limb that returns enzyme expression to baseline as substrate is consumed, rendering the system self-limiting and self-reporting (loop closure). Both arms are co-localized within the plastisphere biofilm at the plastic surface, which stabilizes labile CRISPR components against matrix interference.

To raise the loop above the cascaded isothermal and enzymatic amplification, the primary collateral-cleavage signal is mechanistically nested within secondary amplification chemistries that operate isothermally and therefore remain compatible with field deployment. In the pre-amplification configuration, the released aptamer activator is first multiplied by recombinase polymerase amplification or rolling-circle amplification, increasing the molar concentration of Cas12a-activating species before the collateral-cleavage stage and thereby converting a sub-attomolar recognition event into a saturating activation signal. In the post-amplification configuration, the products of trans-cleavage are funneled into a transduction layer with intrinsic gain where the liberation of an electrochemiluminescent emitter from a metal–organic-framework cage, or the unmasking of a G-quadruplex/hemin DNAzyme that catalytically generates a colorimetric or electrochemical output. The mechanistic consequence of cascading these stages is multiplicative rather than additive gain, as shown in Figure 8.

The proposed mechanistic for signal to command expression of the depolymerizing enzymatic cascade can be in (i) low-power controller that administers a diffusible chemical inducer to a spatially adjacent engineered consortium, thereby derepressing a synthetic operon encoding PETase, MHETase, leaf-branch compost cutinase, or laccase; (ii) intracellularly relayed modality, the polymer-derived hydrolysis intermediates themselves or characteristic leachate small molecules serve as the inducing ligands for an allosteric transcription factor or a riboswitch that drives a positive-feedback amplification of degradative-enzyme synthesis, so that initial low-level depolymerization detected by the sensing arm autocatalytically accelerates further degradation; or (iii) fully integrated modality, a single chassis deploys a catalytically dead dCas12a fused to a transcriptional activation domain whose effective concentration of available crRNA is itself governed by the same aptamer-gated strand-displacement event that drives sensing.

Therefore, the architecture requires a negative-feedback limb that returns degradative gene expression to baseline once the polymer substrate, and hence the recognition signal, is depleted. Mechanistically, this is achievable by making the inducing signal strictly dependent on the continued presence of the substrate or its proximal degradation intermediates: as the aptamer-recognizable polymer surface is consumed, the strand-displacement activator is no longer regenerated, the Cas-activating guide pool collapses, and dCas-mediated transcriptional activation decays at a rate set by the dilution and turnover of the activator and of the enzymes themselves.

## 6. Current Challenges

The field has moved from foundational gene-editing principles toward applied biosensing and bioremediation platforms.

On the detection side, the molecular basis for CRISPR-based MP sensing is now reasonably well established. Coupling polymer-specific DNA aptamers to collateral-cleavage effectors such as Cas12a and Cas13a allows non-nucleic-acid pollutants-PVC and polystyrene among them to be transduced into quantifiable molecular signals.

On the remediation side, the PlastiCRISPR framework has translated microbial plastic degradation into a more controllable and metabolically optimized process. Editing *I. sakaiensis*, *P. putida*, *E. coli*, and *H. bluephagenesis* to enhance PET hydrolase, LCC, FAST-PETase, and PHA-biosynthetic activities through Cas9 brings together enzyme engineering, synthetic biology, and circular-economy thinking. Synthetic microbial consortia add ecological realism and functional breadth. No single organism carries the full metabolic repertoire needed to depolymerize, assimilate, and valorize the chemically and physically diverse mixture of environmental MPs. Taken together, these capabilities suggest a plausible trajectory toward integrated systems in which portable biosensors detect MPs and their leachates, engineered consortia carry out targeted degradation, and plastic-derived carbon is rerouted into useful products within circular frameworks.

These prospects are tempered by challenges that must be resolved before field deployment. The selectivity of DNA aptamers for chemically similar polymer classes has not been rigorously validated in complex environmental matrices that contain competing organic molecules, humic acids, trace metals, surfactants, and dense microbial biomass; aptamer binding affinity and structural stability are known to deteriorate under such conditions. Likewise, the functional stability of CRISPR components, guide RNAs, Cas effectors, and reporters under the variable temperature, pH, salinity, and UV exposure of natural waters, sediments, and soils has not been comprehensively characterized, and losses in these parameters could erode detection sensitivity and remediation efficiency in the field. Finally, the metabolic cost of overproducing degradative enzymes such as PETase and cytochrome P450 manifesting as slower growth, compromised membrane integrity, and lower product yields calls for additional balancing strategies, plausibly including dynamic regulatory circuits that activate degradative genes only when the target substrate is present.

## 7. Future Perspectives and Research Directions

Realizing CRISPR-based detection and degradation of microplastics at environmental scale will require coordinated progress on several fronts. First, aptamer recognition elements must be selected and counter-selected directly in complex matrices so that polymer specificity is retained in the presence of recalcitrant organic compounds (dyes, phenolic compounds, etc.), surfactants, trace metals, and dense microbial biomass [93,94]. Second, the field stability of Cas effectors, guide RNAs, and reporters should be systematically characterized across the temperature, pH, salinity, and UV ranges of real waters, sediments, and soils, and improved through lyophilization, encapsulation, or thermostable Cas variants [95]. Third, the metabolic burden of over-expressing depolymerases calls for dynamic, substrate-gated circuits that switch on degradative genes only when the target polymer is present. Finally, biosafety, biocontainment, long-term genetic stability of engineered strains, and regulatory acceptance must be addressed early, ideally through built-in kill-switches and containment strategies, to enable responsible translation from the laboratory to the environment.

## 8. Conclusions

CRISPR-Cas technologies offer a coherent, mechanistically grounded route to both detecting and remediating microplastics, with detection resting on guide-programmable collateral cleavage and remediation resting on precise metabolic engineering of plastic-degrading microorganisms and consortia. The principal value of an integrated view is that the same molecular logic supports complementary functions across the monitoring mitigation range. Realizing this potential at environmental scale will depend on resolving matrix interference, reagent robustness under field conditions, and host metabolic burden. Progress on these fronts, particularly through stimulus-responsive regulatory circuits, computationally designed allosteric Cas variants, and rigorously validated field assays will determine how far these laboratory advances can be carried into practical environmental technologies. Ultimately, uniting programmable molecular recognition with programmable metabolism offers a rare opportunity to convert one of the most persistent pollutants of our time into a monitorable, degradable, and even valorizable resource, provided the community now invests in field validation, biosafety, and standardization to carry these principles from proof of concept to practice.

## Figures and Tables

**Table 1:** Representative microorganisms engineered for enhanced plastic degradation.

Organism	Product	Pathway/Process	Substrate	Degradation Performance	References
*Streptomyces* spp.	Polyhydroxyalkanoates (PHA)	PET biodegradation	Pre-treated post-consumer PET	+ (slow; secretory)	[37]
*Pseudomonas* spp.	Biopolymers	Hydrolysis and fermentation	Various plastic-related substrates	++ (monomer valorization)	[38]
*Ideonella sakaiensis*	PET monomers	PET degradation	PET	++ (native assimilator)	[39]
*Kineococcus endophyticus* Un-5	PET-degrading enzyme	Genetic engineering and expression	PET	+ (n.r. rate)	[40]
*Rhodococcus* spp.	Hydrolytic enzymes	Biodegradation	Various plastics	+ (broad host)	[41]
*Vibrio alginolyticus*	Hydrolytic enzymes	Biodegradation	Polyvinyl alcohol, LDPE	+ (PVA, LDPE)	[42]

++/+ denote moderate/low; n.r. = not reported.

**Table 2:** Comparison of CRISPR-Cas9 editing and synthetic consortia for plastic degradation.

Technology	Application	Example	Impact	References
CRISPR-Cas9	Gene editing for enzyme optimization	Improved PETase thermostability in *E. coli*	Accelerates degradation across varied conditions	[57]
Synthetic consortia	Engineered microbial communities	*E. coli* + *P. putida* for PET and PU degradation	Broader degradation capability	[58]

**Table 3:** Representative CRISPR/Cas-based platforms reported for microplastic detection.

Target MPs	Recognition Element/Readout	Cas Effector	Sensing Mechanism	LOD	Selectivity in Complex Matrix/Reagent Stability	References
PS	PS-specific aptamer coupled with split gRNA/Electrode	Cas12a	Electrochemical (G-quadruplex/hemin on Au electrode)	45 ng mL^−1^	++ (fouling risk)/++	[85]
PVC	PS-specific aptamer coupled with split gRNA/Electrode	Cas12a	Electrochemical	37 ng mL^−1^	++ (fouling risk)/++	[85]
Microcystin-LR (environmental toxin)	Aptamer + blocker DNA/Fluorescence	Cas12a trans-cleavage of fluorescent reporter	Fluorescence	3 × 10^−6^ µg L^−1^	+++/++	[87]
Microcystin-LR	Aptamer/Strip	Cas12a trans-cleavage	Lateral flow strip	1 × 10^−3^ µg L^−1^	++/+++	[87]
PS	Aptamer/ECL	Cas12a collateral cleavage with Alq_3_@ZIF-8 ECL amplification	ECL	19.8 ng mL^−1^	++ (n.r. in situ)/++	[88]
PVC	Aptamer/ECL	Cas12a collateral cleavage with Alq_3_@ZIF-8 ECL amplification	ECL	14.5 ng mL^−1^	++ (n.r. in situ)/++	[88]
PVC	PVC-specific aptamer + rolling-circle amplification probe/ECL	Cas12a trans-cleavage activity combined with RCA signal amplification	Cathodic ECL	0.20 ng mL^−1^ to 0.20 μg mL^−1^	+ (n.r.)/+ (multi-step)	[89]

+++/++/+ denote high/moderate/low; n.r. = not reported.

## Data Availability

No new data were created or analyzed in this study. Data sharing is not applicable to this article.

## Abbreviations

The abbreviations used throughout this manuscript are defined below.

**MP/MPs**	Microplastic(s)
**PE**	Polyethylene
**PP**	Polypropylene
**PS**	Polystyrene
**PVC**	Polyvinyl chloride
**PET**	Polyethylene terephthalate
**PHA**	Polyhydroxyalkanoate
**CRISPR**	Clustered regularly interspaced short palindromic repeats
**Cas**	CRISPR-associated protein
**crRNA**	CRISPR RNA
**tracrRNA**	Trans-activating CRISPR RNA
**sgRNA**	Single guide RNA
**PAM**	Protospacer-adjacent motif
**DSB**	Double-strand break
**NHEJ**	Non-homologous end joining
**HDR**	Homology-directed repair
**CRISPRi**	CRISPR interference
**ROS**	Reactive oxygen species
**EPS**	Extracellular polymeric substances
**QS**	Quorum sensing
**RPA**	Recombinase polymerase amplification
**LAMP**	Loop-mediated isothermal amplification
**RT-RPA**	Reverse-transcription RPA
**LCC**	Leaf-branch compost cutinase
**ARGs**	Antibiotic-resistance genes
**FTIR**	Fourier-transform infrared spectroscopy
**SHERLOCK**	Specific high-sensitivity enzymatic reporter unlocking
**DETECTR**	DNA endonuclease-targeted CRISPR trans reporter
**PCR**	Polymerase chain reaction
**SNP**	Single-nucleotide polymorphism
**ECL**	Electrochemiluminescence
**RCA**	Rolling-circle amplification
**PETase**	PET hydrolase/PET-degrading enzyme
**MHETase**	Mono-(2-hydroxyethyl) terephthalate hydrolase
**BHET**	Bis(2-hydroxyethyl) terephthalate
**MHET**	Mono-(2-hydroxyethyl) terephthalate
**TPA**	Terephthalic acid
**EG**	Ethylene glycol
**CBM3**	Carbohydrate-binding module 3
**dCas**	Catalytically dead Cas
**PU**	Polyurethane
**LDPE**	Low-density polyethylene
